# Giant Diverticular Surprise

**DOI:** 10.14309/crj.0000000000000642

**Published:** 2021-08-26

**Authors:** Neal Shah, Anthony Razzano, Rani Modayil

**Affiliations:** 1 Department of Internal Medicine, NYU Langone Hospital, Long Island, Mineola, NY; 2 Department of Gastroenterology and Hepatology, NYU Langone Hospital, Long Island, Mineola, NY

## CASE REPORT

**Figure 1.**
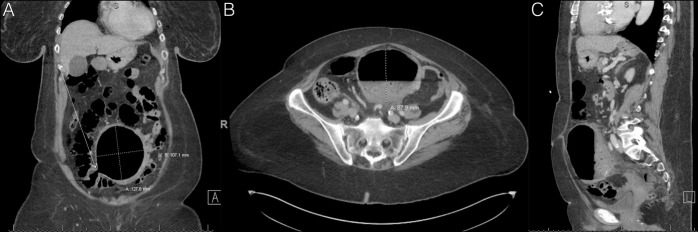
(A) Coronal, (B) transverse, and (C) sagittal view of abdominal and pelvic computed tomography with contrast showing a sigmoid colon with a giant diverticulum.

A 77-year-old woman with a medical history of iron deficiency anemia, previous collapsed vocal cord, and previous diverticular bleed 3 years earlier presented with 2 episodes of painless, bright red blood per rectum requiring multiple transfusions. The patient was febrile, tachycardic, and tachypneic. Abdominal and pelvic computed tomography with contrast (Figure 1) showed a sigmoid colon with 13-cm giant diverticulum with mild wall thickening and inflammatory stranding, suggesting a diagnosis of acute diverticulitis. She was managed conservatively and placed on intravenous ceftriaxone. Her symptoms resolved as she was transitioned to oral antibiotics on discharge. The patient remains stable in the outpatient setting, and a follow-up is planned with surgery for elective sigmoid colectomy.

## DISCLOSURES

Author contributions: All authors contributed equally to this manuscript. N. Shah is the article guarantor.

Financial disclosure: None to report.

Informed consent was obtained for this case report.

